# Antibodies to soluble liver antigen and α-enolase in patients with autoimmune hepatitis

**DOI:** 10.1186/1740-2557-1-4

**Published:** 2004-11-19

**Authors:** Dimitrios-Petrou Bogdanos, Daniele Gilbert, Ilaria Bianchi, Simona Leoni, Ragai R Mitry, Yun Ma, Giorgina Mieli-Vergani, Diego Vergani

**Affiliations:** 1 Institute of Liver Studies, King's College Hospital, Denmark Hill, London SE5 9RS, UK; 2 Faculté de Médecine et Pharmacie, U519 INSERM, University of Rouen, Rouen, France

## Abstract

**Background:**

Antibodies to a cytosolic soluble liver antigen (SLA) are specifically detected in patients with autoimmune hepatitis (AIH). The target of anti-SLA has been identified as a ~50 kDa UGA serine tRNA-associated protein complex (tRNP^(Ser)Sec^), through the screening of cDNA libraries. A recent report questioned the identity of tRNP^(Ser)Sec ^as the real SLA antigen. The latter study identified α-enolase as a major anti-SLA target, through proteomic analysis.

**Methods:**

In an attempt to explain the observed discrepancy we have investigated reactivity of SLA positive sera against α-enolase and tRNP^(Ser)Sec ^using rat and primate liver homogenate and the recombinant antigens. Thirty-three serum samples, 11 from SLA-positive patients and 22 from SLA negative controls were investigated. SLA antibodies were detected by an inhibition ELISA and confirmed by immunoblot using human liver homogenate. Autoantibody reactivity was further evaluated using preparations of primate and rat liver homogenates. Anti-α-enolase antibody reactivity has been tested by immunoblot using recombinant α-enolase. An affinity purified goat polyclonal anti-α-enolase IgG antibody was used as reference serum sample. Anti-tRNP^(Ser)Sec ^antibody reactivity was detected by ELISA or dot blot using recombinant tRNP^(Ser)Sec ^antigen.

**Results and Discussion:**

The affinity purified IgG antibody directed to human α-enolase gave a band of approximately 48 kDa in both human and rat liver homogenates. A high titre anti-tRNP^(Ser)Sec ^antibody serum gave a single band of ~50 kDa in both liver preparations. All but one anti-SLA antibody positive sera reacted with a ~50 kDa but none immunofixed a 48 kDa band. All anti-SLA antibody positive sera reacted strongly with the recombinant full length tRNP^(Ser)Sec ^protein. None of the anti-SLA negative sera reacted with tRNP^(Ser)Sec^. Anti-SLA positive, and anti-SLA negative sera reacted equally against recombinant α-enolase by immunoblot. Pre-incubation of anti-SLA positive sera with tRNP^(Ser)Sec ^completely abolished the 50 kDa band. The findings of the present study indicate that α-enolase and tRNP^(Ser)Sec ^are both expressed in primate and rat liver and have a respective MW of 48 and 50 kDa. They also show that anti-tRNP^(Ser)Sec ^– but not anti-α-enolase – correlates with anti-SLA antibody reactivity.

**Conclusion:**

Our findings indicate that tRNP^(Ser)Sec ^is the most likely target of anti-SLA.

## Background

Antibodies to a cytosolic soluble liver antigen (SLA), detected originally by an inhibition ELISA using cytosolic liver fractions in a sub-group of patients with autoimmune hepatitis (AIH) negative for other autoantibodies, have recently been also reported in adult patients with anti-nuclear and/or smooth muscle antibody (ANA/SMA) positive type 1 AIH and in seronegative patients with a form of cryptogenic hepatitis resembling type 1 AIH [1-6]. In pediatric patients, anti-SLA has been described not only in type 1 AIH but also in anti-liver kidney microsomal-1 antibody positive type 2 AIH and autoimmune sclerosing cholangitis [7-10]. Anti-SLA is specific for these autoimmune liver diseases, where it is associated with a more severe course and is virtually absent in non-hepatic autoimmune disorders [1-9]. The target of anti-SLA has been identified by several groups as a ~50 kDa UGA serine tRNA-associated protein complex (tRNP^(Ser)Sec^), through the screening of cDNA libraries [2-4,7]. Anti-tRNP^(Ser)Sec ^antibodies have been detected in up to 90% of serum samples positive for SLA by the original inhibition ELISA [1-8].

Using anti-SLA positive sera against rat liver cytosolic fraction in one and two-dimensional immunoblotting analyses and through peptide mass fingerprint analysis, following MALDI-TOF mass spectrometry, Ballot et al. [11] identified four isoforms of α-enolase, – a cytosolic antigen of 48–50 kDa –, as the major target of anti-SLA positive sera. These findings challenge the notion that tRNP^(Ser)Sec ^is the sole target of anti-SLA antibodies [2-8]. Critically, no absorption studies were performed with purified α-enolase to confirm this proposal [11]. Moreover, α-enolase has been described as an antigen in several autoimmune disorders totally unrelated to autoimmune hepatitis [12-18].

Using recombinant tRNP^(Ser)Sec ^antigen as competitor in inhibition experiments it has been found removal of the 50 kDa band immunofixed by SLA positive sera from immunoblots of primate liver homogenate [19]. Though this finding indicates tRNP^(Ser)Sec ^as a major component of SLA, a view apparently shared by Ballot et al, several questions still remain unanswered:

1. Are there any differences in α-enolase expression between rat – used by Ballot et al [11] – and primate liver homogenate – used by our study [19] – that could explain the discrepancy between these studies?

2. Is it true that failure of proteomic analysis to detect tRNP^(Ser)Sec ^is due to its presence in trace amounts in the supernatant of liver homogenate [11]?

3. What is the reactivity of SLA positive and negative sera against recombinant α-enolase?

4. How do we explain the apparent paradox of SLA being identified as α-enolase by proteomic analysis and as tRNP^(Ser)Sec ^by the screening of cDNA libraries? Do α-enolase and tRNP^(Ser)Sec ^cross-react?

In the present study, we have investigated reactivity of SLA positive sera against α-enolase and tRNP^(Ser)Sec ^using rat and primate liver homogenate and the recombinant antigens.

## Methods

### Patients

Thirty-three serum samples, 11 from SLA-positive patients and 22 from SLA negative controls were investigated. SLA-positive patients included 8 paediatric patients with AIH1 [7 female, median age 12, range 5–17 years, all ANA positive, median immunofluorescence (IFL) titre: 1/320, range 1/80–1/1280] and 3 adults with AIH/PBC overlap syndrome, (2 female, median age 56, range 47–65), all but one AMA positive (1/5120), and ANA positive (median tire 1/320, range 1/80–1/640). Eleven case-matched SLA negative patients were tested as pathological controls including 8 with AIH1 and 3 with AIH/PBC overlap syndrome. Eleven demographically matched healthy subjects including 8 children (7 female, median age 11, range 6–16) and 3 adults (2 female, median age 53, range 42–63) negative for SLA were also tested as controls.

### Antibody Detection

All sera have been tested for conventional antibodies by indirect IFL using rodent liver, kidney, stomach tissues.

SLA antibodies were detected by a modified inhibition ELISA [1] and confirmed by immunoblot using human liver homogenate [20]. Autoantibody reactivity was further evaluated using preparations of primate (Euroimmun, Lubeck, Germany) and rat (AID Autoimmun   Diagnostika GmbH, Strassberg, Germany) liver homogenates, according to manufacturers' instructions. Anti-α-enolase antibody reactivity has been tested by immunoblot using recombinant α-enolase, as described previously [17]. Briefly, the complete complementary DNA (cDNA) encoding human α-enolase was isolated from a cDNA expression library derived from synoviocytes obtained from a patient with rheumatoid arthritis (Stratagene, La Jolla, CA) and immunoscreened with goat anti-enolase antibodies. This cDNA was subcloned in frame in the pSPUTK in vitro translation vector (Stratagene) using the Apa I and Bam HI restriction sites. The translation product was synthesized as a separated biotinylated polypeptide, purified by SoftLink Soft Release Avidin Resin (Promega, Madison, WI), migrated in SDS-PAGE, according to the method of Laemmli, and electrotransferred onto a nitrocellulose membrane [17]. The filters were then incubated with goat anti-enolase antibodies (see below), a monospecific anti-α-enolase antibody positive serum from a patient with rheumatoid arthritis or with individual serum samples, in Tris buffered saline, 0.05% Tween 20, 5% dry milk for 2 hours. After washing, the filters were incubated for 1 hour with 1:15,000-diluted peroxidase-conjugated goat anti-human IgG (Sigma-Aldrich) in 0.05% TBST-milk. The filters were washed and revealed by a chemiluminescence reaction (Supersignal; Pierce, Rockford, IL) [17].

An affinity purified goat polyclonal anti-α-enolase IgG antibody raised against a peptide mapping near the carboxyl-terminus of human α-enolase, which is common to α, β, and γ isoforms of mouse, rat and human enolase (200 μg/ml; Santa Cruz Biotechnology, Santa Cruz, California, USA) was used as reference serum sample at a dilution of 1:100, according to the manufacturer's instructions.

Anti-tRNP^(Ser)Sec ^antibody reactivity was detected by ELISA or dot blot using recombinant tRNP^(Ser)Sec ^antigen (Euroimmun). A high-titre anti-tRNP^(Ser)Sec ^antibody positive serum was used as a positive control.

### Inhibition Studies

To investigate whether the 50 kDa band immunofixed by anti-SLA is tRNP^(Ser)Sec^, inhibition experiments were performed using 3 anti-SLA positive serum samples, diluted at 1/1000, and pre-incubated with solid phase recombinant tRNP^(Ser)Sec ^(Euroimmun, UK), as previously described [19].

## Results and Discussion

**Figure 1:**
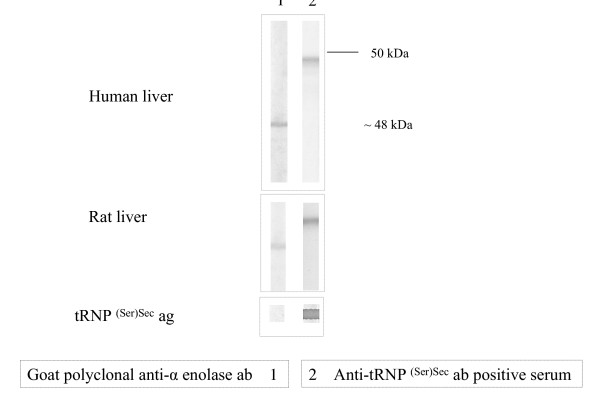
Immunoblot patterns produced by anti-α-enolases and anti-tRNP^(Ser)Sec ^antibodies on electrophoretically separated primate and rat liver homogenates and dot blot results with recombinant tRNP^(Ser)Sec^. In both rat and primate liver preparations, a band of ~48 kDa is immunofixed by a polyclonal goat IgG anti-α-enolase specific antibody; a band of ~50 kDa is immunofixed by a serum containing a high-titre anti-tRNP^(Ser)Sec ^antibody. Anti-α-enolase antibody does not recognize tRNP^(Ser)Sec ^by dot blot analysis.

**Figure 2:**
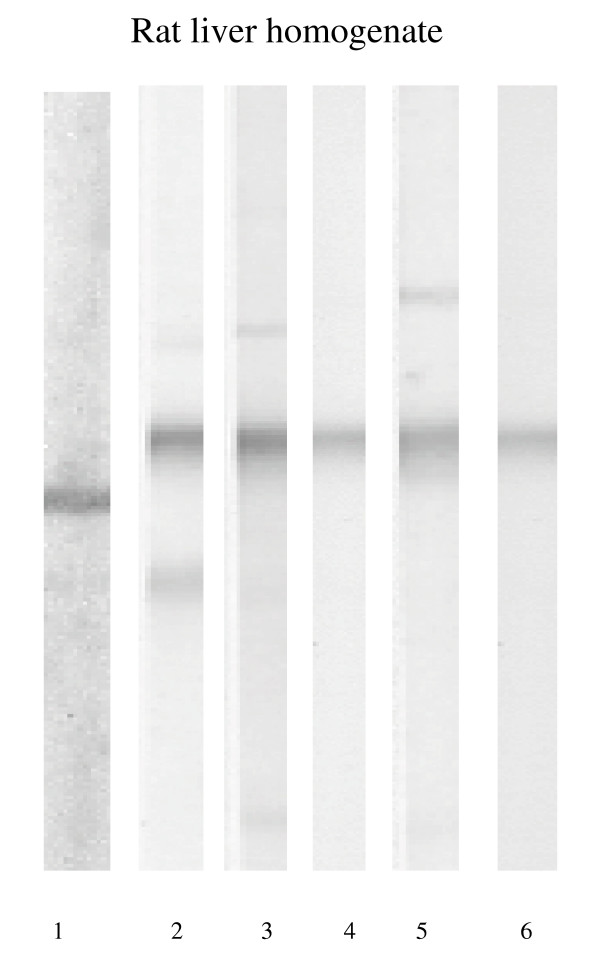
Immunoblot patterns produced on rat liver homogenate by (lane 1) a polyclonal goat IgG anti-α-enolase specific antibody; (lanes 2–5) four representative anti-soluble liver antigen (SLA) positive sera; (lane 6) a reference serum containing high-titre anti-tRNP^(Ser)Sec ^antibody.

**Figure 3:**
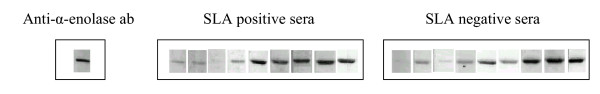
Immunoblot patterns obtained using 9 SLA positive and 9 SLA negative serum samples against recombinant α-enolase. A polyclonal goat IgG anti-α-enolase specific antibody has been used as a reference positive serum. Ab, antibody; ag, antigen

The affinity purified IgG antibody directed to human α-enolase gave a band of approximately 48 kDa in both human and rat liver homogenates as shown in Figure 1. A high titre anti-tRNP^(Ser)Sec ^antibody serum gave a single band of ~50 kDa in both liver preparations (Figure 1). All but one anti-SLA antibody positive sera reacted with a ~50 kDa band similar to that obtained by the high titre anti-tRNP^(Ser)Sec ^antibody serum in rat liver preparations (Figure 2) but none immunofixed a 48 kDa band. All anti-SLA antibody positive sera reacted strongly with the recombinant full length tRNP^(Ser)Sec ^protein both in ELISA (mean titre 87 ± 23 RU/ml, cut off: 20 RU/ml) and dot blot. None of the anti-SLA negative sera reacted with tRNP^(Ser)Sec^. Anti-SLA positive, and anti-SLA negative sera reacted equally against recombinant α-enolase by immunoblot with 5/9 cases in each group giving a strong band (Figure 3).

Pre-incubation of anti-SLA positive sera with tRNP^(Ser)Sec ^completely abolished the 50 kDa band immunofixed in either rat or human liver preparation. In contrast, a parallel experiment where the anti-α-enolase antiserum was pre-incubated with recombinant tRNP^(Ser)Sec ^left unaltered the reactivity to the 48 kDa band.

The findings of the present study indicate that α-enolase and tRNP^(Ser)Sec ^are both expressed in primate and rat liver and have a respective MW of 48 and 50 kDa. They also show that anti-tRNP^(Ser)Sec ^– but not anti-α-enolase – correlates with anti-SLA antibody reactivity suggesting that the target of anti-SLA antibody is tRNP^(Ser)Sec ^and not α-enolase [1-9,19].

However, Ballot et al [11] state that α-enolase is a major SLA antigen since rat α-enolases have MW of 47.4 to 47.5 and Pi values of 5.8 to 6.2. These characteristics do not match the MW (48.8) and Pi (8.6) of tRNP^(Ser)Sec^. Ballot et al [11], however, show a Coomassie stained 2D-gel over the Pi range 6 to 11 and an immunoblot of this gel with several bands of a Pi above 8, but have not investigated these bands by MALDI-TOF analysis being therefore unable to rule out their possible relation to tRNP^(Ser)Sec^.

## Conclusion

All the above observations indicate that tRNP^(Ser)Sec ^is the most likely target of anti-SLA.

## List of Abbreviations

AIH, autoimmune hepatitis; SLA, soluble liver antigen; tRNP^(Ser)Sec^, tRNA-associated antigenic protein

## Competing Interests

The author(s) declare that they have no competing interests.

## Authors' Contributions

DPB designed the study, performed the ELISA and inhibition experiments and contributed in writing the report. DG performed the immunoblot testing of anti-enolase antibodies. IB, SL & YM performed the immunoblot experiments involving liver preparations. RRM helped with the artwork. GMV provided clinical material and contributed in writing the report. DV supervised the study and wrote the report.

## References

[B1] Manns M, Gerken G, Kyriatsoulis A, Staritz M, Meyer zum Buschenfelde KH (1987). Characterisation of a new subgroup of autoimmune chronic active hepatitis by autoantibodies against a soluble liver antigen. Lancet.

[B2] Wies I, Brunner S, Henninger J, Herkel J, Kanzler S, Meyer zum Buschenfelde KH, Lohse AW (2000). Identification of target antigen for SLA/LP autoantibodies in autoimmune hepatitis. Lancet.

[B3] Costa M, Rodriguez-Sanchez JL, Czaja AJ, Gelpi C (2000). Isolation and characterization of cDNA encoding the antigenic protein of the human tRNP(Ser)Sec complex recognized by autoantibodies from patients withtype-1 autoimmune hepatitis. Clin Exp Immunol.

[B4] Wen L, Ma Y, Bogdanos DP, Wong FS, Demaine A, Mieli-Vergani G, Vergani D (2001). Pediatric autoimmune liver diseases: the molecular basis of humoral and cellular immunity. Curr Mol Med.

[B5] Volkmann M, Martin L, Baurle A, Heid H, Strassburg CP, Trautwein C, Fiehn W, Manns MP (2001). Soluble liver antigen: isolation of a 35-kd recombinant protein (SLA-p35) specifically recognizing sera from patients with autoimmune hepatitis. Hepatology.

[B6] Ma Y, Okamoto M, Thomas MG, Bogdanos DP, Lopes AR, Portmann B, Underhill J, Durr R, Mieli-Vergani G, Vergani D (2002). Antibodies to conformational epitopes of soluble liver antigen define a severe form of autoimmune liver disease. Hepatology.

[B7] Vitozzi S, Djilali-Saiah I, Lapierre P, Alvarez F (2002). Anti-soluble liver antigen/liver-pancreas (SLA/LP) antibodies in pediatric patients with autoimmune hepatitis. Autoimmunity.

[B8] Czaja AJ, Shums Z, Norman GL (2002). Frequency and significance of antibodies to soluble liver antigen/liver pancreas in variant autoimmune hepatitis. Autoimmunity.

[B9] Baeres M, Herkel J, Czaja AJ, Wies I, Kanzler S, Cancado EL, Porta G, Nishioka M, Simon T, Daehnrich C, Schlumberger W, Galle PR, Lohse AW (2002). Establishment of standardised SLA/LP immunoassays: specificity for autoimmune hepatitis, worldwide occurrence, and clinical characteristics. Gut.

[B10] Vergani D, Choudhuri K, Bogdanos DP, Mieli-Vergani G (2002). Pathogenesis of autoimmune hepatitis. Clin Liver Dis.

[B11] Ballot E, Bruneel A, Labas V, Johanet C (2003). Identification of rat targets of anti-soluble liver antigen autoantibodies by serologic proteome analysis. Clin Chem.

[B12] Adamus G, Amundson D, Seigel GM, Machnicki M (1998). Anti-enolase-alpha autoantibodies in cancer-associated retinopathy: epitope mapping and cytotoxicity on retinal cells. J Autoimmun.

[B13] Lee KH, Chung HS, Kim HS, Oh SH, Ha MK, Baik JH, Lee S, Bang D (2003). Human alpha-enolase from endothelial cells as a target antigen of anti-endothelial cell antibody in Behcet's disease. Arthritis Rheum.

[B14] Moodie FD, Leaker B, Cambridge G, Totty NF, Segal AW (1993). Alpha-enolase: a novel cytosolic autoantigen in ANCA positive vasculitis. Kidney Int.

[B15] Pratesi F, Moscato S, Sabbatini A, Chimenti D, Bombardieri S, Migliorini P (2000). Autoantibodies specific for alpha-enolase in systemic autoimmune disorders. J Rheumatol.

[B16] Roozendaal C, Zhao MH, Horst G, Lockwood CM, Kleibeuker JH, Limburg PC, Nelis GF, Kallenberg CG (1998). Catalase and alpha-enolase: two novel granulocyte autoantigens in inflammatory bowel disease (IBD). Clin Exp Immunol.

[B17] Saulot V, Vittecoq O, Charlionet R, Fardellone P, Lange C, Marvin L, Machour N, Le Loet X, Gilbert D, Tron F (2002). Presence of autoantibodies to the glycolytic enzyme alpha-enolase in sera from patients with early rheumatoid arthritis. Arthritis Rheum.

[B18] Tanaka S, Tatsumi KI, Takano T, Murakami Y, Takao T, Yamakita N, Tahara S, Teramoto A, Hashimoto K, Kato Y, Amino N (2003). Anti-alpha-enolase antibodies in pituitary disease. Endocr J.

[B19] Bogdanos DP, Bianchi I, Ma Y, Mitry RR, Mieli-Vergani G, Vergani D (2004). Targets of antibodies to soluble liver antigen in patients with autoimmune hepatitis. Clin Chem.

[B20] Bogdanos DP, Pares A, Baum H, Caballeria L, Rigopoulou EI, Ma Y, Burroughs AK, Rodes J, Vergani D (2004). Disease-specific cross-reactivity between mimicking peptides of heat shock protein of mycobacterium gordonae and dominant epitope of E2 subunit of pyruvate dehydrogenase is common in Spanish but not British patients with primary biliary cirrhosis. J Autoimmun.

